# Right Ventricular Free Wall Strain in Healthy Lowlanders and Highlanders—A Case-Control Study

**DOI:** 10.3390/jcm15041548

**Published:** 2026-02-15

**Authors:** Helga Preiss, Talant Sooronbaev, Stéphanie Saxer, Michael Furian, Simon R. Schneider, Maamed Mademilov, Paula Appenzeller, Felix C. Tanner, Konrad E. Bloch, Silvia Ulrich, Mona Lichtblau

**Affiliations:** 1 Clinic of Pulmonology, University Hospital Zurich, 8091 Zurich, Switzerland; helga.preiss@usz.ch (H.P.); stephanie.saxer@usz.ch (S.S.); michael.furian@usz.ch (M.F.); simonrafael.schneider@usz.ch (S.R.S.); paula.appenzeller@usz.ch (P.A.); konrad.bloch@usz.ch (K.E.B.); silvia.ulrich@usz.ch (S.U.); 2 Swiss-Kyrgyz High Altitude Medicine and Research Initiative, Bishkek 720040, Kyrgyzstan; sooronbaev@inbox.ru (T.S.); mademilov@gmail.com (M.M.); 3 National Center for Cardiology and Internal Medicine, Department of Respiratory Medicine, Bishkek 720040, Kyrgyzstan; 4 Department of Health, Republic University of Applied Sciences St. Gallen, 9001 St. Gallen, Switzerland; 5 Swiss-Kyrgyz High Altitude Medicine and Research Initiative, 8091 Zurich, Switzerland; 6 University Heart Center, University Hospital Zurich, University of Zurich, 8091 Zurich, Switzerland; felix.tanner@usz.ch; 7 Department of Cardiology, University Heart Center, University Hospital Zurich, 8091 Zurich, Switzerland

**Keywords:** high-altitude pulmonary hypertension, right ventricular strain analysis, RVFWS

## Abstract

**Background/Objectives**: It is widely acknowledged that healthy highlanders (HL) present with significantly higher pulmonary arterial pressure (PAP) compared to healthy lowlanders (LL). However, whether this elevated PAP solely signifies a response to hypoxia at altitude or is also linked to right ventricular (RV) dysfunction is still unknown. Therefore, we assessed RV function in HL and LL using speckle-tracking-derived strain analysis. **Methods**: This case-control study evaluates echocardiographic RV free wall strain (RVFWS) in LL and HL in Kyrgyzstan. A RVFWS over −20% for men and a RVFWS of −21% for women were considered indicators of RV dysfunction. Subgroup analysis included individuals with and without risk for pulmonary hypertension (PH), defined as a TRV > 2.8 m/s. **Results**: A total of 59 participants (21 LL, 38 HL), with a mean ± SD age of 43 ± 8 versus 48 ± 10 years, were included and assessed at their living altitude. RVFWS in HL and LL was −27.3% ± 4.7 versus −27.0% ± 6.0 (mean difference 0.13%, 95%CI −2.65 to 2.92, *p* = 0.852). The conventional RV indices RV FAC (42% ± 6 vs. 38% ± 8), TAPSE (2.2 cm ± 0.2 vs. 2.0 cm ± 0.3), and TDI S’ (14.2 cm/s ± 1.9 vs. 12.1 cm/s ± 1.8), however, did differ significantly between LL and HL. HL with and without risk for PH did not differ in RVFWS and in the conventional RV indices. **Conclusions**: Despite significant differences in conventional RV markers, healthy highlanders generally did not differ in RVFWS compared with lowlanders, indicating maintained RV systolic function at high altitude. Our findings suggest that elevated PAP in HL reflects adaptation rather than RV dysfunction, underscoring the need for refined diagnostic criteria for clinically relevant high-altitude pulmonary hypertension.

## 1. Introduction

Approximately 140 million individuals worldwide live at high altitude (>2500 m above sea level) [1]. Residing at high altitude poses extreme challenges, necessitating profound physiological adjustments. Hypobaric hypoxia is widely acknowledged as the most daunting impediment to human viability in high-altitude environments. It induces hypoxic vasoconstriction in the lungs, leading to elevated pulmonary artery pressure (PAP). Common adaptation mechanisms of the human organism to high altitude are an increase in ventilation and heart rate, reduced plasma volume, and enhanced erythropoiesis [2]. Recent studies suggest a genetic adaptation across successive generations of individuals habituated permanently at high altitude [3]. But what are the limits of human adaptation to this hypoxic milieu, and how can we diagnose manifestations of cardiac dysfunction due to hypoxia-induced pulmonary hypertension (PH)?

Previous investigations have revealed a significant increase in mean PAP (mPAP) in healthy high-altitude inhabitants in comparison to healthy lowlanders [4,5]. High altitude pulmonary hypertension (HAPH) is defined by mPAP > 30 mmHg or a systolic pulmonary arterial pressure (sPAP) > 50 mmHg [6]. It is estimated that the prevalence of HAPH varies between 6 and 35% according to the definition used [5,7,8] while the prevalence of PH at sea level lies around 1% [9]. But to what extent HAPH is associated with symptoms, prognosis, and impaired right ventricular (RV) function and consequently with an increased morbidity and mortality is unknown [10]. It is widely acknowledged that despite prolonged exposure to hypoxia, no progress of distal vascular remodeling to obliterative, plexiform lesions, characteristic of severe PH, is observed [11,12]. These observations might imply that vascular alterations remain moderate in HAPH. Also, its potential for reversibility distinguishes HAPH from pulmonary arterial hypertension (PAH), as patients with HAPH may undergo a reversal of pulmonary arterial remodeling under normoxic conditions [13,14,15,16,17]. Pratali et al. conducted a study revealing comparable RV contractile reserve during exercise between individuals with chronic mountain sickness (CMS) and healthy high-altitude inhabitants, despite lower RV function values at rest in CMS patients [18]. Similar findings were reported by Ulrich et al., who observed that during exercise, asymptomatic highlanders exhibit higher resting PAP and lower TAPSE/TRPG (tricuspid annular plane systolic excursion to peak tricuspid regurgitant jet velocity ratio), with no significant divergence in exercise-induced changes between healthy lowlanders and highlanders [19]. In a review by Penazola et al. regarding cardiac function at high altitudes, it is suggested that PH primarily represents evidence of incomplete adaptation compatible with normal life at high altitude, rather than being indicative of impaired RV function. Thus, normal pulmonary pressure, defined as mPAP < 20 mmHg, among HA residents would signify complete adaptation [14]. However, studies assessing RV function in these populations have conflicting results. While two studies demonstrated a significant deterioration in traditional echocardiographic RV parameters in high-altitude residents compared to lowlanders [5,20], another failed to identify RV dysfunction using conventional indices [21]. Given that RV dysfunction is associated with increased morbidity and mortality and represents a major public health concern in mountainous regions worldwide [22,23], it is imperative to evaluate and understand the RV function of highlanders.

Speckle tracking strain analysis represents a non-invasive method for assessing RV function, potentially offering greater sensitivity than conventional echocardiographic indices [24]. RV free wall strain (RVFWS) has been linked to all-cause mortality [25] and serves as a robust predictor for cardiovascular events with 90% sensitivity and 69% specificity [26]. This approach provides valuable insights into RV functions and mechanics, characterized by reduced angle dependency and enhanced accuracy [27].

The aim of the current study was to investigate the RV function of both healthy lowlanders and asymptomatic, and thus, considered healthy highlanders using speckle tracking strain analysis, and to compare the results with those obtained from conventional echocardiographic parameters.

## 2. Materials and Methods

### 2.1. Study Design and Participants

This study is a post-hoc analysis of echocardiograms of an existing cohort study (registered at clinicaltrials.gov NCT03165656) in healthy lowlanders, currently living below 800 m, and healthy highlanders, dwelling above 2500 m. The initial cohort trial was conducted between July 2017 and August 2018 at the National Center for Cardiology and Internal Medicine in Bishkek (760 m) and in the Ak-Say region of the Tien Shan mountain region in Kyrgyzstan, at an altitude of 3250 m (Ak-say Cultural Center). The participants share a similar age and are all of Kyrgyz ethnicity. Lowlanders were born and live at <800 m. Highlanders were born and raised >2500 m in Kyrgyzstan and inhabit a high-altitude plateau, ranging from 2500 m to 3600 m.

The exclusion criteria for highlanders included the presence of excessive erythrocytosis (defined as hemoglobin levels > 19 g·dL^−1^ in females and >21 g·dL^−1^ in males), since excessive erythrocytosis markedly alters the hemodynamic properties of the blood, potentially leading to an overestimation of right ventricular dysfunction. Additional exclusions encompassed cardiopulmonary diseases, like coronary heart disease, COPD, or significant smoking history (>20 cigarettes/day).

This study was conducted in accordance with the Declaration of Helsinki, approved by the ethics committee in Kyrgyzstan (01-8/433) and endorsed by the cantonal ethic review board Zurich (2017-00369). All participants gave written informed consent to participate in the study.

### 2.2. Assessments

During the initial trial, a medical history was elicited, a clinical examination was conducted, and vital parameters were measured. Spirometry was performed in accordance with international guidelines. The New York Heart Association functional class was determined.

Two-dimensional echocardiography was performed using real-time, phased array sector scanners (CX50; Philips AG, Horgen, Switzerland). The results of the left ventricular echocardiographic parameters and the conventional measurements assessing right heart function, such as tricuspid annular plane systolic excursion (TAPSE), fractional area change (RV FAC), systolic and mean PAP, and tissue-doppler-derived S` (S`), as well as the clinical baseline parameters, had already been calculated and published [5]. For this sub-analysis, highlanders were divided into two groups, individuals with and without risk of PH, defined as TRV > 2.8 m/s [28].

The strain analysis was performed by a researcher with experience in this technique. Subjects were excluded if there was evidence of systolic left ventricular dysfunction, incomplete ECG (electrocardiogram), or signs of arrhythmia, inadequate quality of echo images, or insufficient frame rate. For the strain analysis, a three-beat, 2-D, digital clip of RV-focused view was transferred to the strain software (TomTec Image Arena Cardiac Performance Analysis, version 4.6, Philips Ultrasound Business Group, Munich, Germany). Strain analysis by speckle-tracking is a semi-automated technique that measures myocardial deformation by tracking speckles like a footprint and tracing the endocardial contours through the cardiac cycle. Right ventricular free wall strain (RVFWS) is defined as the systolic deformation of the right ventricular free wall relative to its original length at end-diastole and is expressed as a negative percentage, indicating that the more negative the value, the better the RV systolic function. RVFWS was chosen as the primary outcome of this analysis since previous studies have already shown that this parameter correlates best with traditional echocardiographic measurements [29]. According to the American Society of Echocardiography, the absolute cut-off for RV impairment lies over −20% for men and over −21% for women [28]. The normal reference value for the age group of our study population lies at −26.8 ± 4.9 [30]. According to the latest literature, women display a more negative and therefore better RVFWS than men [31,32].

### 2.3. Outcomes

The primary outcome of this study was the difference in RVFWS between lowlanders (<800 m) and highlanders (>2500 m).

Secondary outcomes included the difference in RVFWS between highlanders without vs. with risk for PH, the correlation of RVFWS with TRV in lowlanders and highlanders, and the correlation of RVFWS with the traditional echocardiographic parameters of the RV.

### 2.4. Statistical Analyses

After checking data for completeness, mean ± standard deviation (SD) and mean differences (95% confidence intervals [CI]) were computed. Depending on the results of the Shapiro–Wilk test and Levene’s test, differences between the two altitudes and subgroups were calculated either by the Student’s *t*-test, Welch’s *t*-test, or the Wilcoxon rank-sum test, as appropriate. Univariable and multivariable linear regression were performed, including clinical measurements and echocardiographic parameters, to find independent predictors of RVFWS. Statistical significance was assumed at a *p*-value of <0.05 or 95% CI not including zero. The analysis was conducted with R (R Foundation for Statistical Computing, Version 2022.12.0 + 353, Vienna, Austria).

## 3. Results

**Figure 1:**
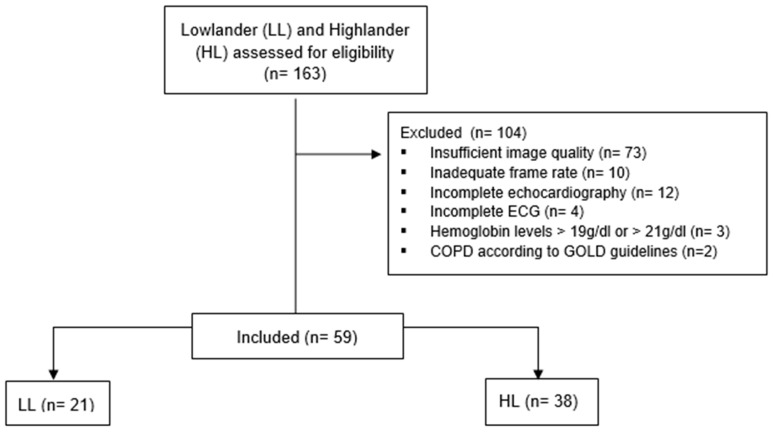
Flowchart.

Out of 163 eligible participants, the echocardiographic data of 59 subjects were available and sufficient for strain analysis (Figure 1). The baseline characteristics are presented in Table 1. Twelve LL (57%) and 16 HL (37%) were male, with an average smoking history of 24 ± 3 pack years across all LL and 19 ± 7 pack-years across all HL (*p* = 0.139). Most of the LL were in NYHA class I (76%) with an average age of 43 ± 8 years, while most of the HL were in NYHA class I (50%) and II (45%) (*p* = 0.060) with an average age of 48 ± 10 years (*p* = 0.061).

We observed a significant difference in heart rate between LL and HL. As expected, the PaO_2_ and the PaCO_2_ were significantly lower in HL, while the hemoglobin and hematocrit levels were higher. While HL and LL did not differ in the 6-min walk test, HL experienced significantly more dyspnea and fatigue after exercise according to the Borg Score. Stroke volume and LVEF were lower in highlanders.

### Right Ventricular Function

**Figure 2:**
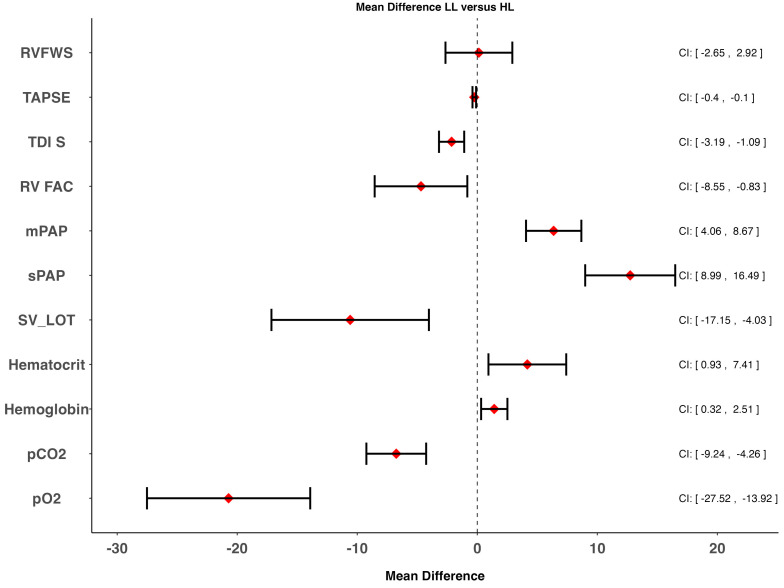
Mean differences of different blood gas parameters and echocardiographic indices of the right ventricle between lowlanders (LL) and highlanders (HL). CI: 95% confidence interval. A CI not crossing 0 is considered statistically significant. RVFWS: right ventricular free wall strain; TAPSE: tricuspid annular plane systolic excursion; TDI: tissue Doppler image; RVFAC: right ventricular fractional area change; mPAP: mean pulmonary artery pressure; sPAP: systolic pulmonary artery pressure; SV_LOT: stroke volume left ventricular outflow tract; pCO_2_: partial pressure of carbon dioxide; pO_2_: partial pressure of oxygen.

**Figure 3:**
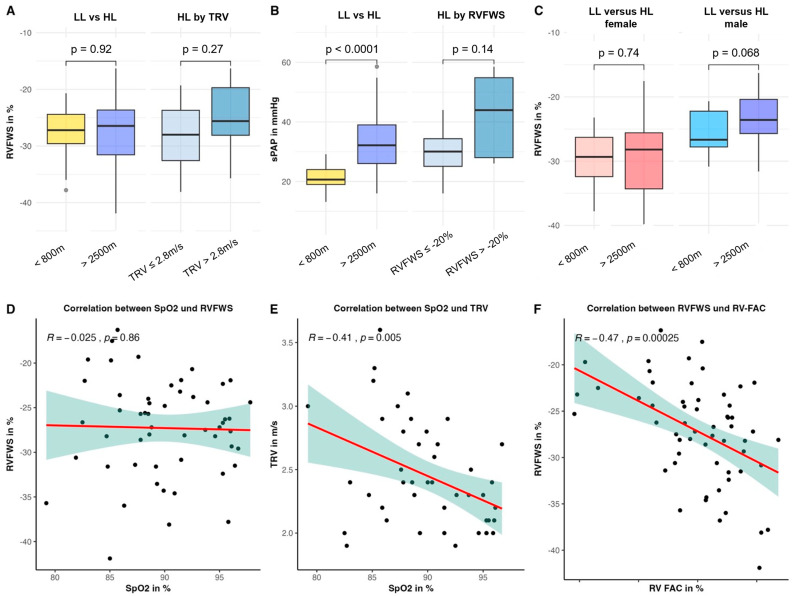
Boxplots and scatterplots of different right ventricular parameters. (**A**) Right ventricular free wall strain (RVFWS) in % in Lowlanders (LL; <800 m) and Highlanders (HL; >2500 m), and in highlanders without risk of PH (TRV ≤ 2.8 m/s) and with risk of PH (TRV > 2.8 m/s). (**B**) Systolic pulmonary arterial pressure (sPAP) in mmHg in LL (<800 m) and HL (>2500 m), and in HL without right ventricular dysfunction (RVFWS < −20%) and with right ventricular dysfunction (RVFWS > −20%). (**C**) Sex specific differences of RVFWS in % between lowlanders (<800 m) and highlanders (>2500 m). (**D**) Overall correlation between RVFWS in % and SpO_2_ in % independent of altitude. (**E**) Overall correlation between TRV and SpO_2_ independent of altitude. (**F**) Overall correlation between RVFWS and RV-FAC independent of altitude.

Results of the RV echocardiographic assessment can be found in Table 2 and Figure 2 and Figure 3. Pulmonary arterial pressures were significantly elevated in HL compared to LL. All the other echocardiographic indices of right heart function (RV FAC, TAPSE, TDI S’) were significantly lower in HL compared to LL, albeit within normal range. RVFWS, however, was similar in healthy LL and in HL (Figure 3A). TAPSE/sPAP and RVFWS/sPAP as surrogates for RV-PA coupling were both reduced between lowlanders and HL. Women had a significantly better RVFWS than men (Figure 3C). According to the linear regression analysis, sex, BMI, e′ lateral, and RV-FAC were moderate independent predictors for RVFWS (Supplementary Table S3, Figure 3C,F). Although these results should be interpreted with caution, since RVFAC and lateral e′ are not pathophysiologically independent from RVFWS, as these parameters capture overlapping components of right ventricular systolic function. There was no correlation between SpO_2_ and RVFWS (*p*= 0.86) (Figure 3D), but as expected, there was a moderate correlation between SpO_2_ and TRV (Figure 3E).

Additionally, we compared HL without and with risk of PH (Table 1 and Table 2), defined as a TRV > 2.8 m/s. HL with risk of PH (TRV > 2.8 m/s) showed a significant decrease in stroke volume (*p* = 0.028). Among right ventricular echocardiographic indices, only the coupling parameters TAPSE/sPAP (*p* < 0.001) and RVFWS/sPAP (*p* < 0.001) significantly differed between HL with and without PH risk. However, this difference is largely attributable to the use of TRV in defining the respective subgroups. All other parameters did not differ between HL without and with the risk of PH.

Individual data revealed 5 (13%) out of 38 HL with RVFWS ≥ −20% (Supplementary Table S1), albeit only 3 out of those 5 had a TRV > 2.8 m/s. HL with RV dysfunction based on RVFWS had no significant difference in sPAP (Figure 3, Panel B, Supplementary Table S2). Additionally, HL with RV impairment due to RVFWS showed significantly lower pO_2_ levels than HL without RV dysfunction (Supplementary Table S2). The left ventricular parameters e′ lateral, e′ septal, and the E/A ratio were all significantly impaired in participants with RV dysfunction assessed by RVFWS (Supplementary Table S2).

HL had no significantly higher proportion of subjects with impaired right ventricular function based on conventional echocardiographic parameters or RVFWS, when compared to LL (Supplementary Table S1).

Moreover, no statistically significant differences in RVFWS were observed between female participants in the LL and HL groups (−30.0% vs. −29.2%; mean difference 0.8%, 95% CI −3.4 to 5.1) or between male participants in the LL and HL groups (−25.7% vs. −22.9%; mean difference 2.8%, 95% CI −0.3 to 5.9), although these results should be interpreted with caution due to limited statistical power.

## 4. Discussion

To the best of our knowledge, this is the first study using RVFWS measured by ultrasound speckle tracking to investigate the prevalence of RV dysfunction in healthy highlanders compared to healthy lowlanders. While traditional echocardiographic parameters of RV function were significantly lower in highlanders compared to lowlanders (albeit within the limits of normal), RVFWS was similar between the two groups. When comparing highlanders with and without risk of PH, defined as a TRV > 2.8 m/s, no significant deterioration in either RVFWS or traditional echocardiographic parameters was observed. Both TAPSE/sPAP and RVFWS/sPAP, markers of RV-pulmonary arterial (RV-PA) coupling, showed significant reductions between lowlanders and highlanders, as well as between highlanders with and without risk of PH. However, these findings should be interpreted with caution, as a rise of sPAP at high altitude is suspected to be physiological, and thus these indirect markers might not be adequate surrogates for RV-PA coupling in a high-altitude population. Notably, e′ lateral, a marker for left ventricular diastolic dysfunction, and RV FAC were both independent predictors of RVFWS, while pO_2_ showed only a significant difference between highlanders with impaired RVFWS compared to subjects with normal RVFWS. Moreover, BMI and sex were identified as independent, moderate predictors of RVFWS, with women exhibiting a significantly lower, and therefore, better RVFWS compared to men.

The discrepant findings between other echocardiographic parameters of the RV function and RVFWS in highlanders can be explained by their differing load dependence and sensitivity. Conventional echocardiographic measures of RV systolic function—such as TAPSE, TDI S′, and RVFAC—are highly sensitive to increases in afterload caused by elevated sPAP [33]. At high altitude, increased sPAP primarily reflects physiological adaptation to chronic hypobaric hypoxia rather than intrinsic RV dysfunction. Although the RV is exposed to higher pressures, it generally preserves or even increases cardiac output through compensatory mechanisms, including increased heart rate. RVFWS is less influenced by loading conditions and more indicative of intrinsic myocardial contractility [31,33]. The superiority of RVFWS for detecting RV dysfunction likely derives from its ability to quantify global RV performance across the entire cardiac cycle, incorporating both systolic and diastolic phases [34]. In contrast, TAPSE, TDI S′, and RVFAC are measures based on a single ventricular dimension [34]. Consequently, no meaningful differences in RVFWS should be expected between healthy LL and healthy HL, as also demonstrated in our study. A recent study by Shima et al. evaluated RV function in patients with lung disease and hypoxia using MRI and right-heart catheterization [35]. They found preserved RV contractility despite impaired RV–pulmonary artery coupling and reduced diastolic dysfunction. Similarly, Kolb et al. demonstrated in chronic lung disease that mild hemodynamic abnormalities—such as elevated pulmonary artery pressure and RV hypertrophy—are common in chronic hypoxia, yet RV contractility is typically preserved. When significant RV dysfunction is present, causes beyond isolated hypoxia should therefore be considered [36]. For chronic hypoxia, long-term oxygen therapy remains the only established treatment option. Consistent with this, the study by Shima et al. demonstrated no therapeutic benefit from pulmonary vasodilator therapy in patients with chronic lung disease and hypoxia [35]. In our cohort, individuals with impaired RVFWS exhibited significantly lower pO_2_ compared with those with normal RVFWS, suggesting that long-term oxygen therapy should be considered in such cases and that alternative causes of RV dysfunction should be explored.

Those findings support the notion that the right ventricle in healthy highlanders remains in a compensated state despite hypoxia through several adaptive mechanisms. Although the stroke volume is reduced in highlanders, total circulating blood volume is significantly increased in native high-altitude populations [37]. Two key adaptive mechanisms account for this phenomenon. First, in compensated highlanders, an increased heart rate maintains a normal cardiac output (CO) despite a reduced stroke volume. Second, higher hemoglobin concentrations enhance oxygen delivery efficiency [38]. Consistent with those known adaptation mechanisms, healthy highlanders in our cohort demonstrated significantly higher heart rates and hemoglobin levels, resulting in preserved CO despite significantly lower stroke volumes. In individuals with significant RV dysfunction, however, CO generally declines, and this reduction is strongly associated with poorer survival [39]. The chronicity of increased afterload also plays a critical role. Acute elevations are more likely to precipitate RV dilation and decompensation, whereas chronic increases allow for adaptive remodeling, in which the RV behaves more similarly to a normal left ventricle. These adaptations are most pronounced when exposure begins at birth, as in high-altitude natives [40].

These observations indicate that elevated sPAP alone is insufficient for defining clinically relevant HAPH and that additional parameters should be integrated into diagnostic criteria. Consistent with this suggestion, the new Guidelines for the Echocardiographic Assessment of the Right Heart from the American Society of Echocardiography emphasize that no single measurement can fully assess right ventricular function or reliably predict outcomes [28]. Because RVFWS is less load-dependent than traditional echocardiographic measures, we suggest this parameter be included alongside conventional indices when evaluating RV function at high altitude. RVFWS remains stable despite moderate elevations in sPAP and decreases only in the presence of significant myocardial dysfunction, which is generally absent in healthy highlanders. A retrospective study by Wright et al. showed that RVFWS provided superior reproducibility and sensitivity compared with RVFAC for sequential RV assessment across a range of PASP values [41]. Furthermore, earlier definitions of HAPH stipulated the absence of excessive erythrocytosis; however, at extreme altitudes, hemoglobin concentrations exceeding 21 g/dL are observed in nearly all individuals [42]. Taken together, these findings underscore the need to reconsider and refine the current definition of HAPH [42].

Interestingly, e′ lateral, e′ septal, and the E/A ratio were all significantly impaired in participants with RV dysfunction assessed by RVFWS. The American Society of Echocardiography emphasizes that RV strain and diastolic markers are jointly influenced by intrinsic myocardial properties and commonly deteriorate in parallel in right-sided heart disease [28]. This reflects the close interplay between systolic impairment, elevated filling pressures, and adverse RV remodeling and might also point to postcapillary PH rather than HAPH. In our study, right ventricular remodeling, as reflected by RV wall thickness, was only mild, further supporting the notion that the highlanders were generally healthy and exhibited preserved, compensated right ventricular function.

The sex- and BMI-related differences in RVFWS observed in our cohort are consistent with population-based data demonstrating more favorable RV strain values in women and reduced strain in individuals with higher BMI [30].

In summary, our study supports the notion that the human heart can successfully adapt to chronic hypoxia without developing myocardial dysfunction, except in rare cases of maladaptation. Nevertheless, the altitude of residence should be considered when interpreting the likelihood of right ventricular impairment. Although the prevalence of pulmonary hypertension remains low even at extremely high altitudes, the likelihood of its development increases with greater altitude of residency [42]. Quantifying RV function with 2-dimensional echocardiography remains challenging due to the RV’s irregular shape, retrosternal position, notable interobserver variability, and the dependence of traditional indices on loading conditions and imaging angle. Prospective and interventional studies are needed to further refine our understanding of RV mechanics at high altitude.

### Limitations

Since this was a post-hoc analysis of a previously conducted study, the echocardiography was not specifically focused on strain analysis. Moreover, the absence of verification of the echocardiographic parameters using the right heart catheter, the gold standard, makes the interpretation of the results more difficult. Finally, the relatively small sample size of our study may reduce the statistical power and limit the generalizability of the findings, especially in regard to the subanalysis of patients with elevated TRV and reduced RVFWS.

## 5. Conclusions

Despite significant differences in conventional echocardiographic markers of RV function, healthy highlanders, living between 2500 and 3600 m, generally exhibit preserved RVFWS compared with lowlanders, indicating maintained RV systolic function at high altitude. Our findings suggest that elevated sPAP in highlanders reflects primarily physiological adaptation rather than RV dysfunction, underscoring the need for refined diagnostic criteria for clinically relevant HAPH.

## Figures and Tables

**Table 1:** Patient characteristics, vital signs, arterial blood gas analysis, exercise capacity, and left ventricular echocardiographic parameters of lowlanders and highlanders without risk of pulmonary hypertension (TRV ≤ 2.8 m/s) and with risk of pulmonary hypertension (TRV > 2.8 m/s).

	Lowlander N = 21	Highlander N = 38	*p*-Value	HL with TRV ≤ 2.8 m/s N = 23	HL with TRV > 2.8 m/s N= 9	*p*-Value
**Patient characteristics**						
Sex			0.219			1
Males	12 (57%)	16 (37%)		8 (35%)	3 (33%)	
Females	9 (43%)	24 (63%)		15 (65%)	6 (67%)	
Age (years)	43 ± 8	48 ± 10	0.061	48 ± 11	50 ± 8	0.558
BMI (kg/m^2^)	28 ± 4	26 ± 5	0.065	25 ± 4	27 ± 3	0.147
Smoking, pack-years	24 ± 3	19 ± 7	0.139	19 ± 1	20 ± 0	0.469
FEV_1_ (Liters)	3.5 ± 0.8	3.1 ± 0.7	0.093	3.1 ± 0.8	2.8 ± 0.5	0.525
FVC (Liters)	4.2 ± 1.0	3.9 ± 0.9	0.254	3.5 ± 0.7	4.0 ± 1.0	0.281
FEV_1_/FVC	82 ± 5	79 ± 4	0.031	78 ± 3	81 ± 5	0.281
NYHA functional class			0.060			1
I	16 (76%)	19 (50%)		12 (52%)	4 (44.5%)	
II	3 (14%)	17 (45%)		11 (48%)	4 (44.5%)	
III	2 (9%)	2 (5%)		0	1 (11%)	
IV	0	0		0	0	
**Clinical parameters**						
Systolic blood pressure (mmHg)	127 ± 19	129 ± 18	0.648	129 ± 18	131 ± 19	0.772
Diastolic blood pressure (mmHg)	81 ± 11	86 ± 11	0.159	85 ± 11	86 ± 12	0.830
Heart rate (beats·min^−1^)	78 ± 9	85 ± 12	0.019	85 ± 12	82 ± 15	0.665
**Arterial blood gas analysis**						
Partial pressure of oxygen (mmHg)	77 ± 13	56 ± 6	< 0.001	59 ± 6	54 ± 5	0.054
Partial pressure of carbon dioxide (mmHg)	40 ± 5	33 ± 3	< 0.001	32 ± 3	35 ± 3	0.058
Hemoglobin (g/dL)	14.4 ± 1.8	15.8 ± 2.2	0.018	15.6 ± 1.7	15.4 ± 2.9	0.837
Hematocrit (%)	42.4 ± 5.3	46.6 ± 6.5	0.019	45.8 ± 5.1	45.2 ± 8.4	0.851
**Exercise Capacity**						
6-min-walk-test (6 MWT in m)	524 ± 89	531 ± 66	0.757	537 ± 55	514 ± 92	0.498
Borg-Dyspnea-Score (at the end of 6 MWT)	0.5 ± 2	3 ± 2	< 0.001	3 ± 2	4 ± 1.5	0.323
Borg-Fatigue-Score (at the end of 6 MWT)	1 ± 3	3 ± 2	0.012	3 ± 2	3 ± 2	0.259
**Left ventricular echocardiographic parameters**						
e′ septal in cm/s	9.6 ± 2.6	8.5 ± 2.1	0.096	8.4 ± 2.1	8.5 ± 2.4	0.939
e′ lateral in cm/s	12.6 ± 3.4	11.6 ± 3.3	0.289	11.4 ± 2.9	11.9 ± 4.5	0.729
E/e′	7.2 ± 2.4	6.4 ± 2.0	0.113	6.5 ± 1.7	6.1 ± 2.5	0.682
E/A	1.2 ± 0.4	1.1 ± 0.4	0.195	1.2 ± 0.5	0.9 ± 0.3	0.052
LA Volume Index (mL/m^2^)	21.5 ± 4.9	18.9 ± 5.1	0.070	19.0 ± 5.1	19.4 ± 6.1	0.872
LVEF biplan (%)	61 ± 4	58 ± 5	0.002	59 ± 5	56 ± 3	0.088

Data are presented as n, n (%), means ± SD. HL: Highlander; BMI: Body Mass Index; FEV_1_: forced expiratory volume in 1 s; FVC: forced vital capacity; NYHA: New York Heart Association Classification. e′ septal: early diastolic myocardial velocity of the mitral annulus at the septal side, measured by tissue Doppler imaging (TDI); e′ lateral: early diastolic myocardial velocity of the mitral annulus at the lateral side; E/e′ : ratio of peak early diastolic transmitral inflow velocity to early diastolic mitral annular velocity; E/A: ratio of early diastolic transmitral flow velocity to late diastolic transmitral flow velocity; LA: left arial. LVEF: left ventricular ejection fraction; TRV: tricuspid regurgitation velocity.

**Table 2:** Right ventricular echocardiographic parameters of lowlanders and highlanders, and highlanders without risk of PH (TRV ≤ 2.8 m/s) and with risk of PH (TRV > 2.8 m/s).

	Lowlander N = 21	Highlander N = 38	*p*-Value	HL with TRV ≤ 2.8 m/s N = 23	HL with TRV > 2.8 m/s N= 9	*p*-Value
**RV strain parameters**						
RV free wall strain (%)	−27.3 ± 4.7	−27.0 ± 6.0	0.852	−27.8 ± 5.6	−25.0 ± 6.4	0.269
RVFWS/sPAP	−1.3 ± 0.3	−0.9 ± 0.3	<0.001	−1.0 ± 0.2	−0.6 ± 0.2	<0.001
**Right ventricular (RV) traditional echocardiographic indices**						
Systolic pulmonary artery pressure (mmHg)	21 ± 4	34 ± 10	<0.001	27 ± 5	43 ± 8	<0.001
Mean pulmonary artery pressure (mmHg)	15 ± 3	21 ± 6	<0.001	19 ± 3	28 ± 5	<0.001
TRV (m/s)	2.1 ± 0.2	2.6 ± 0.4	<0.001	2.4 ± 0.2	3.1 ± 0.2	0.001
Stroke volume (mL)	65 ± 13	55 ± 9	0.002	58 ± 9	50 ± 8	0.028
Cardiac Output (L·min^−1^)	4.4 ± 0.9	4.0 ± 0.8	0.104	4.1 ± 0.7	3.7 ± 1.0	0.289
Right atrial pressure (mmHg)	3 ± 0	4 ± 2	0.559	3 ± 0	3 ± 4	0.559
Right atrial area (cm^2^)	13.5 ± 2.2	13.5 ± 3.4	0.941	13.3 ± 3.7	13.4 ± 3.3	0.972
RV anterior wall diameter (cm)	0.4 ± 0.1	0.4 ± 0.1	0.269	0.4 ± 0.1	0.4 ± 0.1	0.718
RV fractional area change (%)	43 ± 6	38 ± 8	0.027	40 ± 7	35 ± 9	0.094
TAPSE (cm)	2.2 ± 0.2	2.0 ± 0.3	0.001	2.0 ± 0.4	1.9 ± 0.2	0.469
TDI tricuspid annular systolic velocity (cm·s^−1^)	14.2 ± 1.9	12.1 ± 1.8	<0.001	11.7 ± 1.4	12.4 ± 1.8	0.335
TAPSE/sPAP	1.0 ± 0.3	0.7 ± 0.2	<0.001	0.8 ± 0.2	0.5 ± 0.1	<0.001

Data are presented as n, means ± SD; HL: highlander; RV: right ventricular; RVFWS: right ventricular free wall strain; TAPSE: tricuspid annular plane systolic excursion; TDI: tissue Doppler image; sPAP: systolic pulmonary artery pressure. TRV: tricuspid regurgitation velocity.

## Data Availability

The datasets generated and analyzed during the current study are available from the author on reasonable request.

## Supplementary Materials

The following supporting information can be downloaded at: https://www.mdpi.com/article/10.3390/jcm15041548/s1, Supplementary Table S1: Number of individuals with right ventricular impairment according to different echocardiographic parameters and number of highlanders with risk of pulmonary hypertension due to TRV and right ventricular impairment; Supplementary Table S2: Patient characteristics, vital signs, arterial blood gas analysis, exercise capacity and echocardiographic parameters of highlanders with and without RVFWS Impairment; Supplementary Table S3: Linear regression analysis of predictors of RVFWS independent of the living altitude (LL and HL, N = 59).
