# Supplementary material for: Right Ventricular Free Wall Strain in Healthy Lowlanders and Highlanders—A Case-Control Study
Source: J Clin Med. 2026 Feb 15;15(4):1548. doi: 10.3390/jcm15041548 (PMC12942162; doi:10.3390/jcm15041548)
Supplement: Supplementary file 1 [file jcm-15-01548-s001.zip › jcm-4120767-supplementary.pdf]

Supplementary Material

**Supplementary Table S1 Number of individuals with right ventricular impairment** according to different echocardiographic parameters and number of highlanders with risk of pulmonary hypertension due to TRV and right ventricular impairment						
	**Lowlander**	**Highlander**	**p-value**	**HL with TRV ≤ 2.8m/s**	**HL with TRV > 2.8 m/s**	**p-value**
**RVFWS ≥ -20%**	0 (0%)	5/38 (13%)	0.211	2/23 (9%)	3/9 (33%)	0.236
**TAPSE < 1.7cm**	0 (0%)	6/38 (16%)	0.147	4/22 (18%)	1/9 (11%)	1
**TDI S' < 9.5cm/sec**	0 (0%)	3/38 (8%)	0.505	2/23 (9%)	1/9 (11%)	1
**RV-FAC < 35%**	3/21 (14%)	9/36 (25%)	0.535	4/22 (18%)	4/9 (44%)	0.287
Data are presented as n(%). P-value was calculated by Pearson`s Chi-squared test with Yates'continuity. RVFWS: Right ventricular free wall strain; TAPSE: tricuspid annular plane systolic excursion; TDI S': TDI tricuspid annular systolic velocity; RV-FAC: right ventricular fractional area change; TRV: peak tricuspid regurgitation velocity.						

**Supplementary Table S2** Patient characteristics, vital signs, arterial blood gas analysis, exercise capacity and echocardiographic parameters of highlanders with and without RVFWS Impairment			
	**HL with RVFWS < -20 % N= 33**	**HL with RVFWS ≥ -20% N = 5**	**p-value**
**Patients characteristics**			
Age (years)	48 ± 11	50 ± 7	0.583
BMI (kg/m^2)	26 ± 5	26 ± 2	0.722
**Clinical parameters**			
Systolic blood pressure (mmHg)	129 ± 19	127 ± 7	0.706
Diastolic blood pressure (mmHg)	86 ± 12	86 ± 8	0.996
Heart rate (beats·min^{-1})	85 ± 12	83 ± 17	0.826
**Exercise Capacity**			
6-minutes-walk-test (6MWT in m)	526 ± 68	558 ± 50	0.257
Borg-Dyspnea-Score (at the end of 6MWT)	3 ± 2	4 ± 2	0.837
Borg-Fatigue-Score (at the end of 6MWT)	3 ± 2	3 ± 1	0.199
**Arterial blood gas analysis**			
Partial pressure of oxygen (mmHg)	57 ± 6	52 ± 3	0.006
Partial pressure of carbon dioxide(mmHg)	33 ± 3	33 ± 3	0.874
Hemoglobin (g/dl)	15.8 ± 2.3	16.2 ± 1.6	0.634

Hematocrit (%)	46.4 ± 6.8	47.4 ± 4.7	0.702
**Left ventricular echocardiographic parameters**			
e` septal in cm/sec	8.6 ± 2.2	7.4 ± 0.7	0.031
e` lateral in cm/sec	11.9 ± 3.4	9.4 ± 1.8	0.034
E/e`	6.4 ± 2.2	6.0 ± 0.7	0.342
E/A	1.2 ± 0.4	0.9 ± 0.2	0.022
LA Volume Index (ml/m^2)	19.2 ± 5.1	17.1 ± 5.4	0.446
LVEF biplan (%)	58 ± 5	57 ± 2	0.687
**Right ventricular (RV) traditional echocardiographic indices**			
Systolic pulmonary artery pressure (mmHg)	32 ± 9	42 ± 15	0.209
Mean pulmonary artery pressure (mmHg)	20 ± 4	28 ± 9	0.137
TRV (m/s)	2.8 ± 0.5	2.5 ± 0.3	0.122
Stroke volume (ml)	55 ± 9	53 ± 12	0.719
Cardiac Output (L·min^{-1})	4.0 ± 0.7	3.9 ± 1.2	0.769
Right atrial area (cm2)	13.3 ± 3.5	14.9 ± 2.9	0.308
RV anterior wall diameter (cm)	0.4 ± 0.1	0.4 ± 0.1	0.974
RV fractional area change (%)	39 ± 8	33 ± 8	0.074
TAPSE (cm)	2.0 ± 0.3	1.9 ± 0.1	0.118
TDI tricuspid annular systolic velocity (cm·s^{-1})	12.1 ± 1.8	11.8 ± 1.7	0.722
TAPSE/sPAP	0.7 ± 0.2	0.5 ± 0.2	0.058

Data are presented as n(%). P-value was calculated by Pearson`s Chi-squared test with Yates'continuity. BMI: Body Mass Index; e` septal: early diastolic myocardial velocity of the mitral annulus at the septal side, measured by tissue Doppler imaging (TDI); e` septal: early diastolic myocardial velocity of the mitral annulus at the septal side, measured by tissue Doppler imaging (TDI); e` lateral: early diastolic myocardial velocity of the mitral annulus at the lateral side; E/e': ratio of peak early diastolic transmitral inflow velocity to early diastolic mitral annular velocity; E/A: ratio of early diastolic transmitral flow velocity/ late diastolic transmitral flow velocity; LA: left arial. LVEF: left ventricular ejection fraction; TRV: tricuspid regurgitation velocity. RVFWS: Right ventricular free wall strain; TAPSE: tricuspid annular plane systolic excursion; TDI S': TDI tricuspid annular systolic velocity; RV-FAC: right ventricular fractional area change; TRV: peak tricuspid regurgitation velocity.

Supplementary Table S3 Linear regression analysis of predictors of RVFWS independent of the living altitude (LL and HL, N = 59)

**Dependent: RVFWS**	**Univariable**	**Multivariable**
**General characteristics**		
Age	−0.0 (−0.2 to 0.1, p=0.651)	
Sex	−5.1 (−7.7 to −2.6, p<0.001)	−3.6 (−6.1 to −1.2, p= 0.005)
BMI	−0.5 (−0.8 to −0.2, p=0.003)	−0.3 (−0.6 to −0.0, p=0.027)
NYHA 2	−2.9 (−5.9 to 0.1, p=0.056)	
**Exercise Test and Dyspnea Scores**		
6MWT (m)	0.0 (−0.0 to 0.0, p=0.091)	

Borg-Dyspnea-Score (at the end of 6MWT)	−0.0 (−0.8 to 0.8, p=0.942)	
Borg-Fatigue-Score (at the end of 6MWT)	−0.5 (−1.2 to 0.1, p=0.128)	
**Clinical parameters**		
Diastolic BP (mmHg)	0.0 (−0.1 to 0.2, p=0.639)	
Systolic BP (mmHg)	0.0 (−0.1 to 0.1, p=0.583)	
FEV_1 (% predicted)	0.0 (−0.1 to 0.2, p=0.538)	
FEV_1/FVC	−0.0 (−0.4 to 0.3, p=0.791)	
**Arterial blood gases and hemodynamics**		
pO2 (mmHg)	−0.0 (−0.1 to 0.1, p=0.776)	
pCO2 (mmHg)	−0.1 (−0.4 to 0.1, p=0.300)	
Hemoglobin (g/dl)	0.4 (−0.3 to 1.1, p=0.218)	
Hematocrit (%)	0.1 (−0.1 to 0.4, p=0.232)	
Heart rate (beats·min^{-1})	−0.0 (−0.1 to 0.1, p=0.851)	
Stroke volume (ml)	0.0 (−0.1 to 0.2, p=0.488)	
CO (L·min^{-1})	−0.0 (−1.7 to 1.7, p=0.978)	
**Left ventricular diastolic function**		
e` septal in cm/sec	−0.4 (−1.0 to 0.9, p=0.184)	
e` lateral in cm/sec	−0.6 (−1.0 to −0.1, p=0.009)	−0.4 (−0.7 to −0.0, p=0.050)
E/e`	−0.1 (−0.5 to 0.9, p=0.663)	
E/A	−0.4 (−4.1 to 3.2, p=0.809)	
LA Volume Index (ml/m2)	−0.2 (−0.5 to 0.1, p=0.152)	
**Right ventricular traditional echocardiographic indices**		
sPAP (mmHg)	0.0 (−0.1 to 0.2, p=0.473)	
mPAP (mmHg)	0.1 (−1.1 to 0.4, p=0.313)	
TRVmax (m/s)	1.1 (−2.7 to 4.9, p =0.565)	
RAP (mmHg)	−1.2 (−5.7 to 3.3, p=0.593)	
RV wall (in cm)	−2.1 (−12.5 to 16.7, p=0.774)	
Right atrial area (cm2)	0.2 (−0.2 to 0.7, p=0.314)	
TAPSE (cm)	−1.4 (−6.1 to 3.3, p=0.584)	
TDI S’ (cm·s^{-1})	−0.6 (−1.3 to 0.1, p=0.096)	
RV FAC (%)	−0.3 (−0.5 to −0.2, p<0.001)	−0.3 (−0.4 to −0.1, p=0.001)
TAPSE/sPAP	−1.2 (−6.1 to 3.8, p=0.636)	

Data are presented as coefficient, 95%- confidence interval and p. Adjusted R-squared: 0.301; F-statistic: 9.771; p < 0.001. RVFWS: right ventricular free wall strain; RV: right ventricular; BMI: Body Mass Index; NYHA: New York Heart Association Classification; 6MWT: 6 minutes walking test; BP: blood pressure; FEV1: forced expiratory volume in 1s; FVC: forced vital capacity; pO2: partial pressure of oxygen; pCO2: partial pressure of carbon dioxide; CO: cardiac output; e` septal: early diastolic myocardial velocity of the mitral annulus at the septal side, measured by tissue Doppler imaging (TDI); e` lateral: early diastolic myocardial velocity of the mitral annulus at the lateral side; E/e’: ratio of peak early diastolic transmitral inflow velocity to early diastolic mitral annular velocity; E/A: ratio of early diastolic transmitral flow velocity/ late diastolic transmitral flow velocity; LA: left arial. LAVI: left atrial volume index; sPAP: systolic pulmonary artery pressure; mPAP: mean pulmonary artery pressure; TRV: peak tricuspid regurgitation velocity; RAP: Right atrial pressure; RV: right ventricular; TAPSE: tricuspid annular plane systolic excursion; TDI: tissue Doppler image; TDI S’: TDI tricuspid annular systolic velocity; FAC: fractional area change.
* RVFWS is 0.3 units lower in females compared to males.
